# Dexmedetomidine Reduces Diabetic Neuropathy Pain in Rats through the Wnt 10a/*β*-Catenin Signaling Pathway

**DOI:** 10.1155/2018/9043628

**Published:** 2018-11-27

**Authors:** Jun-Min Zhong, Yue-Cheng Lu, Jing Zhang

**Affiliations:** Department of Gynecology and Obstetrics, Guangzhou Women and Children's Medical Center, Guangzhou Medical University, Guangzhou 510623, China

## Abstract

Diabetic neuropathy pain (DNP), a spontaneous pain with hyperalgesia and allodynia, greatly compromises patients' quality of life. Our previous study suggested that dexmedetomidine (DEX) can relieve hyperalgesia in rats by inhibiting inflammation and apoptosis at the level of the spinal cord. In the present study, we aimed to evaluate the role of Wnt 10a/*β*-catenin signaling in DEX-induced alleviation of DNP in rats. Forty-eight rats were randomly allocated to four groups (n=12/group): control, DNP, DEX, and yohimbine groups. The DNP model was established by streptozotocin (STZ) injection. The effects of DEX with or without the *α*_2_ adrenergic antagonist yohimbine were assessed by behavior tests (mechanical withdrawal threshold and thermal withdrawal latency). Spinal cord tissue was evaluated by immunofluorescence staining of astrocytes as well as for Wnt 10a and *β*-catenin expression, western blot analysis of Wnt 10a and *β*-catenin expression, and enzyme-linked immunosorbent assay measurement of proinflammatory cytokines (tumor necrosis factor-*α* and interleukin-1*β*). Rats with STZ-induced DNP had a decreased pain threshold, activated astrocytes, increased expression of Wnt 10a and *β*-catenin, and increased levels of proinflammatory cytokines compared to the control group, and these effects were ameliorated by treatment with DEX. Yohimbine administration partly abolished the protective effects of DEX in the DNP model rats. In conclusion, DEX alleviated DNP in rats by inhibiting inflammation and astrocyte activation, which may be attributed to downregulation of the Wnt 10a/*β*-catenin signaling pathway.

## 1. Introduction

Many diabetes patients suffer from a common chronic complication known as diabetic neuropathy pain (DNP) [1]. Its characteristic symptoms include spontaneous pain, hyperalgesia, and allodynia. It has been reported that this chronic pain is associated with the activation of glial cells and local neuroinflammation [2]. Astrocytes, a type of glial cells, are extensively activated in chronic neuropathy pain and release a large amount of inflammatory mediators that regulate the transmission of pain information [2, 3]. In addition, astrocytes play an important role in the later stage, also known as the maintenance stage, of chronic pain. During this stage, inhibiting the activation of astrocytes can effectively relieve the pain [4]. Therefore, targeting the astrocytes may have some therapeutic potential in DNP treatment. Recently, the activation of the Wnt pathway was found to promote neuroinflammation in the spinal cord and affect neuronal synaptic plasticity [5]. In neuropathy pain, Wnt 10a mediates the activation of classical *β*-catenin signaling, accounting for pain occurrence [6]. However, it is unclear whether the Wnt 10a/*β*-catenin signaling pathway is involved in DNP treatment.

Dexmedetomidine (DEX), a highly selective *α*_2_ adrenergic agonist, exerts anti-inflammatory effects by regulating the balance between the sympathetic [7] and parasympathetic nerve systems [8]. In our previous study, DEX could relieve hyperalgesia and inhibit apoptosis of the spinal cord cells in a rat model of DNP. We also found that DEX inhibited glia activation and inflammatory reactions [9]. However, the underlying anti-inflammatory mechanism of DEX's actions in the treatment of DNP remains to be fully elucidated.

The present study aimed to assess the role of Wnt 10a/*β*-catenin signaling in the effects of DEX in a rat model of DNP. By revealing the specific mechanisms by which DEX inhibits glial activation and the inflammatory response, the results of this study can support further mechanistic studies as well as clinical applications of DEX in the treatment of DNP.

## 2. Materials and Methods

### 2.1. Animals

Male Sprague–Dawley rats (6–8 weeks old, 180–200 g) were obtained from Guangdong Medical Laboratory Animal Center (No. SCXK 2013-0002; Foshan, Guangdong, China). The study protocol was approved by Guangzhou Medical University's Animal Ethics Committee.

Rats were individually housed at a room temperature of 23–25°C without limit of access to food and water. A total of 48 rats were randomly allocated to four groups (n=12 in each group): a control group, a DNP model group (DNP group), a DEX-treated group (DEX group), and a yohimbine-treated group (YOH group). Yohimbine is a selective *α*2-adrenoceptor antagonist that blocks the active site of DEX.

The rat DNP model was established as previously described [9]. For all rats, the fasting period was 16 h for food and 4 h for water. In the three groups that used DNP model rats, the DNP, DEX, and YOH groups, a dose of 70 mg/kg of streptozotocin (STZ, Sigma-Aldrich, USA) was injected intraperitoneally once a day for 3 consecutive days, whereas saline was injected in the control group. At day 3, the level of fasting glucose was measured in a caudal vein blood sample. Type I diabetes was confirmed when by a glucose level ≥16.7 mmol/L. At day 21, the mechanical withdrawal threshold (MWT) was measured, and an MWT <4 g considered indicative of successful establishment of the DNP model. In the DEX group, a dose of 50 *μ*g/kg DEX (Hengrui Medicine Co., Ltd., Jiangsu, China) [9] was injected intraperitoneally once a day for 7 consecutive days from day 28. In the YOH group, rats received intraperitoneal injection of 0.1 mg /kg yohimbine (Sigma-Aldrich) 30 min after DEX administration.

### 2.2. Behavioral Tests

The behavioral tests included the MWT test for the mechanical stimuli response and the thermal withdrawal latency (TWL) test for the thermal stimuli response on days 29–45 after STZ injection.

For the WMT test, we placed the rats in a Plexiglas chamber and used the von Frey filaments (Stoelting Co., USA) to stimulate rats' plantar surface of the left hind paw. Each stimulation lasted for 3–5 s. A quick hind paw withdrawal was defined as a positive response. A force of 0.6 g was applied at the beginning, followed by ascending or descending consecutive stimuli; the force was reduced after a positive response and increased after a negative response. The minimal threshold force to induce the withdrawal response was finally determined. Notably, the force should not exceed 15 g to avoid tissue damage. We calculated the average MWT values from the results of 10 tests to get the 50% withdrawal threshold.

For the TWL test, the thermal stimuli response of the left hind paw was measured using a full-automatic plantar analgesia system (BME-410C, Youer Scientific Co., Shanghai, China). Rats were placed on a 3-mm-thick glass plate within a Plexiglas chamber. Then, the left hind paw was exposed to a heat stimulus directly from a fixed distance. The elapsed time for withdrawal of the paw from the heat was recorded. The average value of five tests performed at 5-min intervals was calculated. A maximum cut-off value of 30 s was used to prevent possible damage.

### 2.3. Tissue Preparation

On days 39, 42, and 45, 350 mg/kg of 10% chloral hydrate was injected intraperitoneally and 250 mL normal saline was used for heart perfusion in rats (n=4 in each group). The spinal cord from L3–5 was isolated and stored.

### 2.4. Western Blot Analysis

Tissues were homogenized, and proteins were extracted in lysis buffer (Beyotime, P0013, China) on ice. After sodium dodecyl sulfate (SDS)-polyacrylamide gel electrophoresis (PAGE), proteins were transferred to a polyvinylidene fluoride membrane. After blocking for 4 h, membranes were incubated with primary antibodies including a rabbit/anti-rat Wnt 10a (1:1000, ab106522; Abcam, USA) and a rabbit/anti-rat *β*-catenin (1:1000, mAb#8480; CST, USA) with a specific buffer (Hiboled Co., HF00088, China) at 4°C overnight. After that, a horseradish peroxidase-conjugated goat/anti-rabbit secondary antibody (1:1000, Santa Cruz Biotechnology) was used applied for incubation for 2 h at room temperature. GAPDH (1:10000, KC-5G5; KangCheng Bio-tech, China) was set as a loading control. The expression bands of target proteins were analyzed with the Quantity One software (version 4.6.2; Bio-Rad Co., USA), and the densitometric values were used for analysis. Normalized protein expression levels were calculated relative to GAPDH expression levels.

### 2.5. Immunofluorescence Staining

Spinal cord segments were fixed and cut into frozen sections. Primary antibodies including a mouse/anti-rat primary antibody against glial fibrillary acidic protein (GFAP) (1:200, ab7260; Abcam, USA), a rabbit/anti-rat Wnt 10a (1:100, ab106522; Abcam, USA), and a rabbit/anti-rat/*β*-catenin (1:100, mAb#8480; CST, USA) were applied for incubation at 4°C overnight. Then, sections were incubated with a biotinylated donkey/anti-mouse or anti-rabbit IgG (1:200; Vector Labs, Burlingame, CA, USA) in 1.5% donkey serum (Jackson Immuno Research Labs, West Grove, PA, USA) for 20 min at 37°C. Finally, a mixture of Alexa Fluor 532- and 488-conjugated secondary antibodies was added for incubation.

A mixture of 50% glycerin in phosphate-buffered saline (PBS) was used to treat the sections. Images were recorded using a confocal laser scanning microscope (Leica SP2, Wetzlar, Germany). The mean fluorescence intensity for GFAP, Wnt 10a, and *β*-catenin was acquired from eight randomly selected sections and calculated with the ImageJ software (National Institutes of Health, Bethesda, MD, USA). The quantification analysis was carried out by an independent researcher unaware of the treatments.

### 2.6. Proinflammatory Factor Levels

Enzyme-linked immunosorbent assay (ELISA) kits (Boster Biological Technology Co., Wuhan, China) were used to measure the proinflammatory factor levels including the tumor necrosis factor (TNF)-*α* and interleukin (IL)-1. The tissues were prepared using a grinder and an ultrasonic tissue homogenizer. The tissue supernatant was collected from centrifuged samples for analysis. The optical density (OD) values were assessed with a microplate reader (NK3; Ladsystems, Helsinki, Finland) at 490 nm. The TNF-*α* and IL-1 levels were determined according to the kit directions.

### 2.7. Statistical Analysis

All statistical analyses were performed with SPSS software (version 21.0, SPSS Inc., Chicago, IL, USA). Data are presented as the mean ± standard deviation (SD) and were compared with one-way analysis of variance, followed by the least significant difference test. Statistical significance was set at a* P*<0.05.

## 3. Results

**Figure 1:**
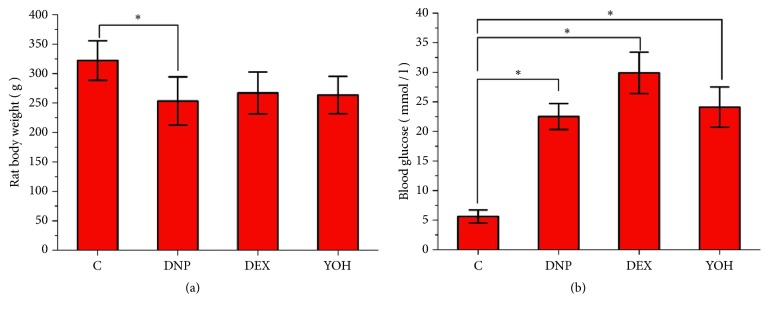
**Body weight and blood glucose levels in rats**. Body weight (a) and blood glucose level (b) on the day 28 after STZ injection. n=12. *∗P*<0.05 for comparisons shown. C, control; DNP, diabetic neuropathy pain; DEX, dexmedetomidine; YOH, yohimbine.

As shown in Figure 1, symptoms of diabetes including polydipsia, polyphagia, and polyuria were observed in the model rats after STZ injection. The body weight and fasting blood glucose levels in the DNP, DEX, and YOH groups were significantly higher than those in the control group. However, body weight and blood glucose did not differ significantly among the three treatment groups (Figure 1), which indicates that the changes in these values after model establishment were not influenced by blood glucose only.

**Figure 2:**
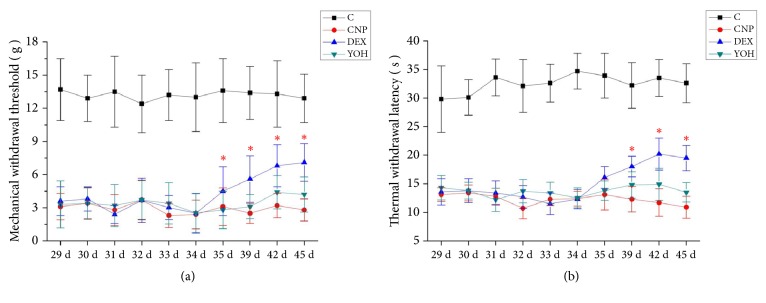
**Behavioral tests.** Mechanical withdrawal threshold (a) and thermal withdrawal latency (b) values on days 29–45 after STZ injection. n=12 for days 29–39; n=8 for day 42; n=4 for day 45. *∗P*<0.05* versus* the DNP group. C, control; DNP, diabetic neuropathy pain; DEX, dexmedetomidine; YOH, yohimbine.

On the behavior tests, the MWT and TWL values were significantly lower in the DNP group than in the control group at all-time points. The DEX group had higher WMT values at days 35–45 and higher TWL values at days 39–45 compared to the control group, but these effects were not seen in the YOH group. These results suggest that DEX alleviated pain in DNP rats by activating the *α*2-adrenoceptor (Figure 2).

**Figure 3:**
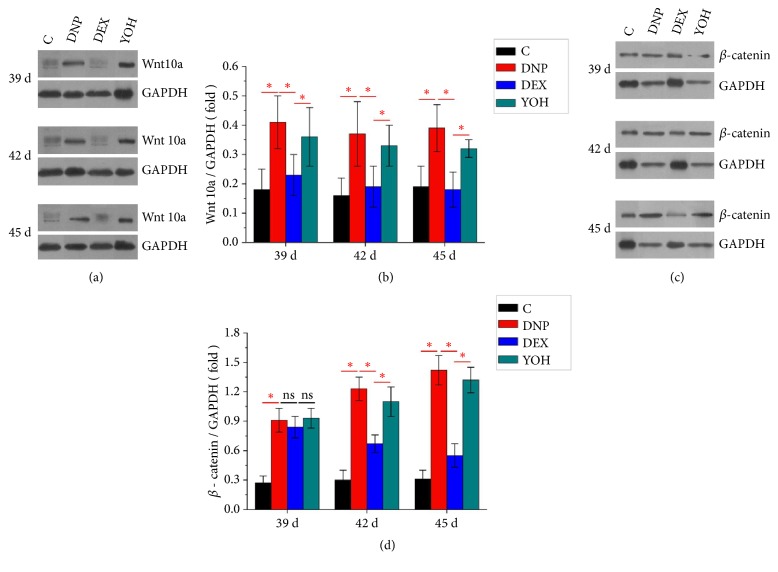
**Western blot analysis showing the protein expression of Wnt 10a and** **β** **-catenin in the spinal cord. **(a, b) Wnt 10a and (c, d) *β*-catenin protein expression normalized to GAPDH expression on days 39, 42, and 45 after STZ injection. n=4. *∗P*<0.05 for comparisons shown. C, control; DNP, diabetic neuropathy pain; DEX, dexmedetomidine; YOH, yohimbine.

As shown in Figure 3, compared to that in the control group, Wnt 10a and *β*-catenin expression in the rat spinal cord was increased in the DNP group. In the DEX group, the protein expression levels of Wnt 10a and *β*-catenin were significantly reduced. However, YOH partly reversed the effect of DEX on Wnt 10a and beta-catenin expression. These data indicate that DEX inhibited the activation of Wnt 10a/*β*-catenin signaling in the spinal cord in the DNP rats through the *α*-2 receptors.

**Figure 4:**
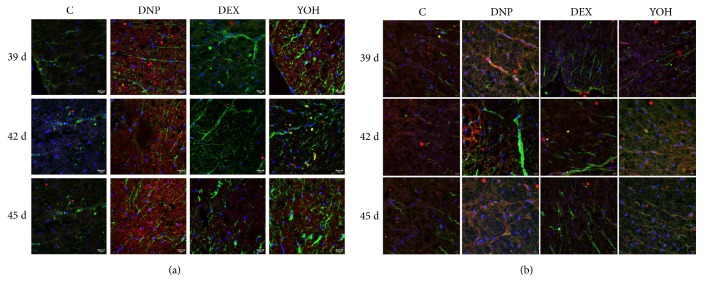
**Immunohistochemical analysis showing astrocyte activation and Wnt 10a and** **β** **-catenin localization.** On days 39, 42, and 45, spinal cord sections were stained with anti-GFAP (green) antibody and anti-Wnt 10a (red) antibody (a). Nuclei were counterstained with DAPI. On days 35, 42, and 45, sections were stained with anti-GFAP (green) antibody and anti-*β*-catenin (red) antibody (b). n=4. C, control; DNP, diabetic neuropathy pain; DEX, dexmedetomidine; YOH, yohimbine.

Compared to those in the control group, the astrocytes in the spinal cord of the DNP group were markedly activated with strong expression of Wnt 10a and *β*-catenin. Activation of astrocytes was inhibited in the DEX group, as was the expression of Wnt 10a and *β*-catenin. Yohimbine reversed the above effects of DEX (Figure 4). Thus, these data indicate that DEX may inhibit astrocyte activation by blocking the Wnt 10a/*β*-catenin signaling pathway.

**Figure 5:**
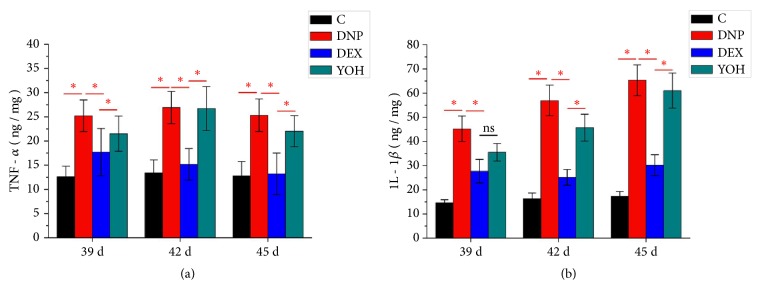
**ELISA showing inflammation in the spinal cord. **(a) TNF-*α* and (b) IL-1*β* levels on days 39, 42, and 45 after STZ injection. n=4. *∗P*<0.05 for comparisons shown. C, control; DNP, diabetic neuropathy pain; DEX, dexmedetomidine; YOH, yohimbine.

As shown in Figure 5, the levels of TNF-*α* and IL-1*β*, activated by the Wnt 10a/*β*-catenin signaling pathway, were significantly increased in the DNP group at days 39, 42, and 45 after STZ injection. Treatment with DEX reduced the levels of TNF-*α* and IL-1*β*, whereas additional treatment with yohimbine reversed the anti-inflammatory effects of DEX. These results indicate that DEX inhibited the inflammatory response within the spinal cord in DNP model rats through *α*2 receptors.

## 4. Discussion

Our present study for the first time demonstrated that dexmedetomidine reduced rat diabetic neuropathy pain, at least in part, via regulating the Wnt-10a signaling pathway. We used a STZ-induced DNP rat model in the present study. STZ is a chemical inducer of diabetes that targets the islet beta cells, causing toxicity including oxidation and hydroxylation. This model is widely used to achieve clinical features of DNP. Our results showed that blood glucose increased and MWT and TWL values decreased in the DNP group from day 29 after STZ injection. In addition, the changes in pain behavior, such as claudication, foot lifting, increased foot licking, and spontaneous neighing, suggested successful establishment of the rat DNP model.

Activation of the classical Wnt/*β*-catenin pathway triggers neuropathy pain. Specifically, the transcriptional regulatory factor, Yes-associated protein (YAP)/transcriptional coactivator with the PDZ-binding motif (TAZ), integrates into *β*-catenin and regulates the Wnt pathway response. Studies have shown that blocking or knocking down YAP/TAZ expression in the spinal cord reduces neuronal damage and Wnt-induced mechanical hyperalgesia [10]. In addition, the nonclassical Wnt/Ryk signaling pathway can promote neuropathy pain in rats through the regulation of neuronal excitability and spinal synaptic plasticity [11]. In the rat models of neuropathy pain and cancer pain, Wnt in the spinal cord is up-regulated rapidly and permanently, activating Wnt/*β*-catenin signaling in the astrocytes and elevating inflammatory cytokine levels [5]. A recent study identified kindlin-1 as a potential therapeutic target for neuropathic pain, for which Wnt-2a, Wnt-3a, Wnt-5a, and Wnt-10a may be the specific ligands [12]. Consistently, Wnt-10a, as a key target, can regulate inflammatory cytokines by regulating *β*-catenin downstream. A recent study found that DEX inhibited the nuclear translocation and binding activity of activated NF-*κ*B, thus reducing inflammatory cytokines [13]. Our results further demonstrated that after DEX treatment, the expression of Wnt-10a was inhibited, *β*-catenin expression was suppressed, and the secretion of inflammatory cytokines was reduced.

Glial cells are the largest group of cells in the nervous system, and they include astrocytes, microgliacytes, NG2 (nerve/glia antigen 2) cells, and oligodendrocytes. In the central nervous system, astrocytes provide neurotrophic support and are important regulators of neurotransmitter functions. Astrocytes, together with microglia, play an important role in neuroinflammation and various neuropathophysiological signal processes. On one hand, intercellular substance exchange happens between the glial cells through functional syncytia formed by gap junctions and neurons. On the other hand, neuronal activity and synaptic plasticity are regulated by the intracellular calcium concentration, the adenylate cyclase-cAMP delivery system, and a remote cell–cell signaling [14]. In normal conditions, the synapses of the resting microglia are closely connected with the axons of neurons, both of which are trimmed by phagocytosis to ensure the stability of axons and synapses [15, 16]. Pathologic conditions will directly lead to a significant increase in the density of immature dendrites [17]. Our previous study also confirmed that DEX attenuates DNP in rats by regulating the microglia.

In the spinal dorsal horn, astrocytes and microglia are activated upon many forms of stimulation including infection, inflammatory mediators, ischemia, apoptosis, and mechanical compression or injury. These activated cells in turn release large amounts of inflammatory cytokines, nerves growth/trophic factors, and neurotransmitters [18]. The activation of glial cells in the spinal cord contributes to neuronal plasticity by releasing inflammatory cytokines and endogenous morphine [19], and the pain-sensing neurons are directly or indirectly activated, resulting in the sensitization of pain neurons [20]. Moreover, the degree of glial cell activation is closely related to the occurrence, development, and duration of hyperalgesia [21]. In the present study, DEX reduced DNP by inhibiting the activation of astrocytes, which was associated with the inhibition of Wnt 10a/*β*-catenin signaling. Thus, the use of DEX may block the positive feedback regulation between the glial cells and inflammatory responses.

In our previous study, hyperalgesia was noted in DNP rats with the activation of microglia but not astrocytes. However, astrocytes were significantly activated in the present study. This difference is due to the different time points for observation. Microglia is significantly activated in the initial stage of chronic pain, followed by the activation of astrocytes [12]. The activated microglia participates in the maintenance of chronic pain. In contrast with the previous study [9], we investigated the effects of DEX at several time points up to day 45 after STZ injection. With a longer observation time, the activation of astrocytes was detected in the present study. One limitation of our current study is the lack of Wnt 10a overexpression or knockdown experiments with or without DEX treatment. Therefore, the current results only provide evidence of changes in Wnt 10a/*β*-catenin signaling in association with DEX treatment. The underlying mechanisms need to be investigated in the future.

In summary, DEX reduced DNP in rats by inhibiting inflammation and astrocyte activation, possibly through the Wnt 10a/*β*-catenin signaling pathway.

## Data Availability

The datasets generated and analyzed during the present study are available from the corresponding author on reasonable request.
